# The role of apoptosis in early embryonic development of the adenohypophysis in rats

**DOI:** 10.1186/1746-160X-4-13

**Published:** 2008-07-23

**Authors:** Jens Weingärtner, Kristina Lotz, Andreas Faltermeier, Oliver Driemel, Johannes Kleinheinz, Tomas Gedrange, Peter Proff

**Affiliations:** 1 Department of Anatomy and Cell Biology, Ernst Moritz Arndt University Greifswald, Friedrich Löffler Straße 23c, D-17487 Greifswald, Germany; 2 Department of Gynecology and Obstetrics, Ernst Moritz Arndt University Greifswald, Wollweberstr. 1, D-17487 Greifswald, Germany; 3 Department of Orthodontics, University of Regensburg, F.J. Strauss-Allee 11, D-93042 Regensburg, Germany; 4 Department of Oral and Maxillofacial Surgery, University of Regensburg, F.J. Strauss-Allee 11, D-93042 Regensburg, Germany; 5 Department of Oral and Maxillofacial Surgery, University of Münster, Waldeyerstraße 30, D-48129 Münster, Germany; 6 Department of Orthodontics, Preventive and Pediatric Dentistry, Ernst Moritz Arndt University Greifswald, Rotgerberstr. 8, D-17489 Greifswald, Germany

## Abstract

**Background:**

Apoptosis is involved in fundamental processes of life, like embryonic development, tissue homeostasis, or immune defense. Defects in apoptosis cause or contribute to developmental malformation, cancer, and degenerative disorders.

**Methods:**

The developing adenohypophysis area of rat fetuses was studied at the embryonic stage 13.5 (gestational day) for apoptotic and proliferative cell activities using histological serial sections.

**Results:**

A high cell proliferation rate was observed throughout the adenohypophysis. In contrast, apoptotic cells visualized by evidence of active caspase-3, were detected only in the basal epithelial cones as an introducing event for fusion and closure of the pharyngeal roof.

**Conclusion:**

We can clearly show an increasing number of apoptotic events only at the basic fusion sides of the adenohypophysis as well as in the opening region of this organ. Apoptotic destruction of epithelial cells at the basal cones of the adenohypophysis begins even before differentiation of the adenohypophyseal cells and their contact with the neurohypophysis. In early stages of development, thus, apoptotic activity of the adenohypophysis is restricted to the basal areas mentioned. In our test animals, the adenohypophysis develops after closure of the anterior neuroporus.

## Background

The adenohypophysis (Rathke pouch) is derived from the ectoderm and develops during the embryonic stage in the pharyngeal roof in front of the pharyngeal membrane before the anterior neuroporus closes. According to Starck (1975), the primordial Rathke pouch (saccus hypophysealis) is a transverse depression in the pharyngeal roof abutting the bottom of the diencephalon without interposed mesenchymal cells [1]. Later, the pouch loses connection with the pharyngeal roof, while a multitude of mesenchymal cells moves between the pharygeal roof and the bottom of the brain [2]. These mesenchymal cells later differentiate into the primordia of the cranial base. The cells of the adenohypophysis proliferate toward the bottom of the brain and further differentiate into hormone-secreting cells. An epithelial bridge may persist between the closed adenohypophysis and the pharyngeal roof (canalis craniophanryngeus) for a longer period. Occasionally, in 2% of the cases [1], this connection develops to the persisting form of a pharyngeal roof hypophysis [3].

Apoptosis is involved in fundamental processes of life, like embryonic development, tissue homeostasis, or immune defense. Defects in apoptosis cause or contribute to developmental malformation, cancer, and degenerative disorders. Apoptosis can be induced in response to many external stimuli (extrinsic pathway) including activation of death receptors such as tumor-necrosis factor (TNF)-receptor 1 or Fas/CD95 by interaction with their cognate ligands [4,5]. Alternatively, various sensors of cellular stress receive signals, for example after DNA damage or growth factor deprivation leading to mitochondrial release of cytochrom c and other apoptogenic factors [6]. Both pathways converge on a cascade system of proteases, called caspases (cysteinyl aspartic proteinases). Activated caspases are the central initiators and executioners of the apoptotic program. Cellular apoptotic events as a consequence of programmed physiological cell death are histomorphologically identifiable by characteristic features such as cell shrinkage, membrane blebbing, and condensed and fragmented nuclear chromatin.

## Methods

38 fetuses from pregnant LEW.1A-rats were collected by caesarean section on day 13.5 of gestation. The fetuses were fixed in 4% buffered formalin solution for 24 hours, embedded in paraffin, and serial frontal sections (5 μm) of the heads were stained with haematoxylin and eosin (HE). Immunohistochemistry on dewaxed and rehydrated sections was performed with the Vectastain Universal Quick kit (Vector Laboratories) according to manufacturer's protocol, and as discribed by *Lotz *et al. (2004) [7]. To determine apoptotic cell death caspase-3 activity was detected by an antibody that specifically binds to the cleaved and thereby activated form form of caspase-3 (Anti-ACTIVE Caspase-3 pAb; Promega, Cleaved Caspase-3 (Asp 175) Ab; Cell Signaling). Proliferating cells were detected using Anti-Rat Ki-67 Ab (MIB-5; DakoCytomation) as primary antibody after heat-induced epitope retrieval in 0.01 M Citrate buffer pH 6.0. Immune complexes were visualized with diaminobenzidine tetrahydrochloride precipitates, and the sections were subsequently counterstained with nuclear fast red (Vector Laboratories).

## Results

At day 13.5 of embryonic development, the primordial adenohypophysis of the rat embryo presents as a cup-like indentation of the pharyngeal roof and, hence, originates from the ectoderm.

Regarding the development of the remaining cranial area, it may be mentioned that the development of the primary nasal ducts is largely completed and the lamina oronasalis at the end of the duct is not yet open. Thus, neither the nasal septum nor a primary palate has developed at this time. Moreover, the maxillary bulges have not yet fused with the nasal bulges, and the nasolacrimal duct between the maxillary bulge and the frontonasal bulge is partly open and not yet completely closed.

The developmental stage outlined here is in agreement with Keibel's (1937) findings [8], however, in his experiments involving Rattus norwegicus Erxleben this stage was reached on the 12^th ^embryonic day, while a connection between the adenohypophysis and the pharyngeal roof no longer existed on day 13.5.

**Figure 1:**
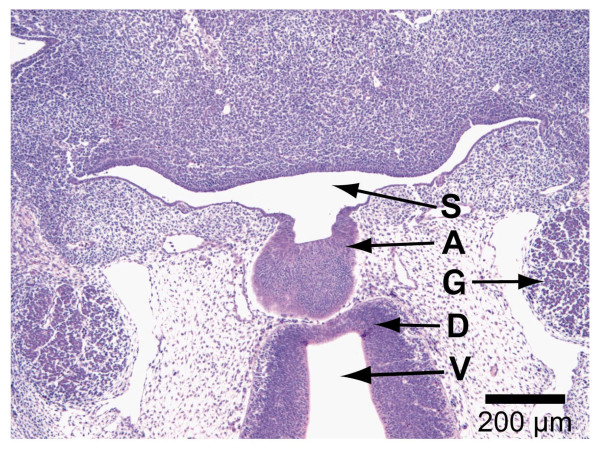
**H&E staining of paraffin section (4 μm), Overview showing: A = adenohypophysis, S = stomatodeum, G = ganglion trigeminale, D = diencephalon, V = 3^rd ^ventricle, 2**.3×.

Moreover, the trigeminal ganglion is clearly noticeable on both sides of the primordial adenohypophysis. Above the adenohypophysis, the diencephalon with the 3^rd ^ventricle is located (Fig. 1).

**Figure 2:**
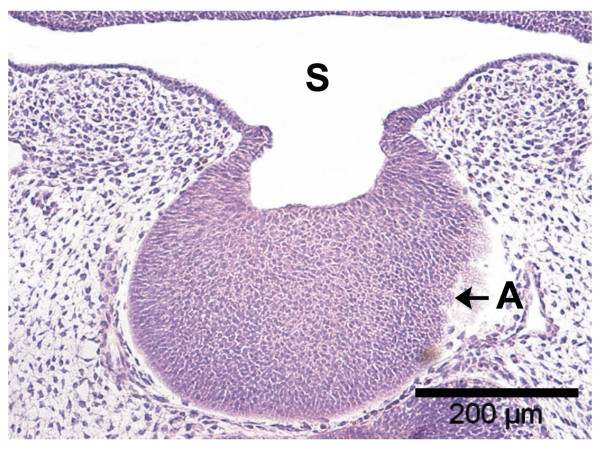
H&E staining of paraffin section (4 μm) through adenopharyngeal area with basally located horizontal epithelial cones and marginal high-prismatic cells: S = stomatodeum, A = adenohypophysis, 10×.

**Figure 3:**
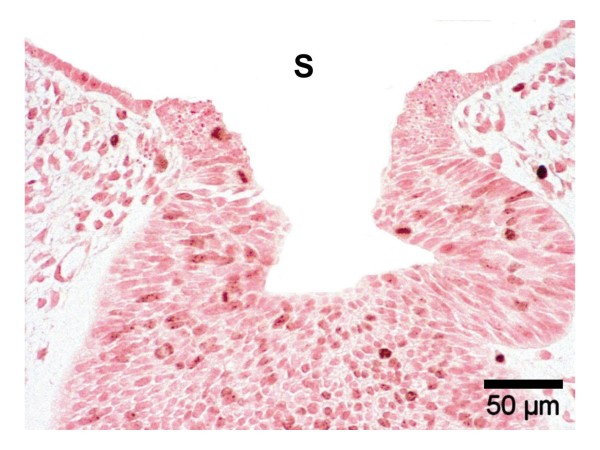
Immunochemistry on paraffin section (4 μm), Ki-67 positive cells in the Rathke pouch and surrounding mesenchyme, but not in the basal epithelial cones, S = stomatodeum, 20×.

In Figure 2, the base of the adenohypophysis and the lumen of this primordial gland are shown in magnification. At the interface with the stomadeal ectoderm two opposite horizontal epithelial cones are noticed whose distance represents the pharyngeal opening of the Rathke pouch and amounts to 100 μm at maximum. A vertical plug running toward the stomadeum as described by Hinrichsen (1993) [9] in a SEM image was not detected in our specimens. Ki-67 marking (Fig. 3) reveals distinct proliferative processes both of the epithelial cells of the adenohypophysis and the surrounding mesenchymal cells. Particularly numerous proliferative cells were noticed in the multi-layered cell assembly and the lateral walls of the Rathke pouch, but not at its base, i.e., the cone-shaped interface with the oral cavity ectoderm. Thus, the marked proliferation of the mesenchymal cells at both sides of the adenohypophyseal base appears to exert lateral pressure on this primordial gland.

**Figure 4:**
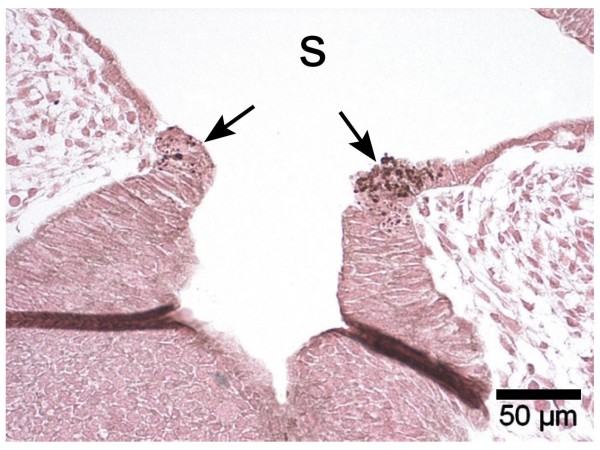
Immunihistochemistry on paraffin section (4 μm) using antibody against active caspase-3 visualizes apoptotic events in the basal epithelial cones of the Rathke pouch (arrows), S = stomatodeum, 20×.

In contrast, the epithelial cells of the adenohypophyseal base and at the pharyngeal roof show a strong apoptotic activity around the bulge visualized by immunohistochemical evidence of active caspase-3 (Fig. 4). A similarly strong activity was not found in the remaining areas of the developing adenohypophysis.

**Figure 5:**
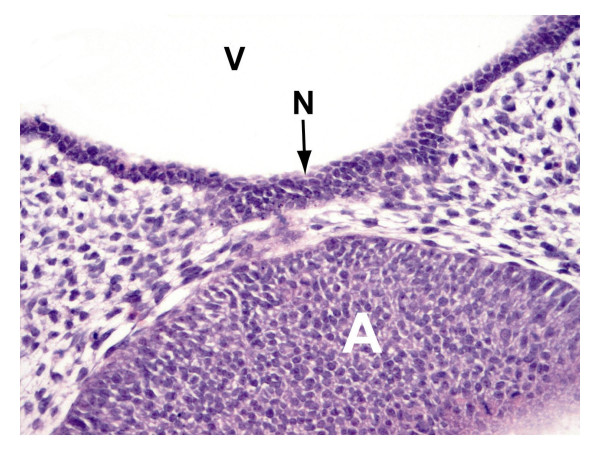
H&E staining of paraffin section of cranial part of the adenohypophysis (A) and vis-à-vis, proliferating primordial neurohypophysis (N), 3^rd ^ventricle (V), 20×.

Figure 5 shows the cranial part of the adenohypophysis proliferating toward the diencephalon. Fewer mesenchymal cells are noticeable directly between the adenohypophysis and the diencephalon. Moreover, an increase of neuroectodermal cells is found at the base of the diencephalon.

## Discussion

Histological serial sections of rat fetuses on day 13.5 of gestation were analyzed for proliferative and apoptotic cell activities during embryonic development of the adenohypophysis. Marked cell division was observed in the cranial area, whereas apoptotic processes were revealed primarily in the basal cells of the adenohypophysis. The process of pharyngeal roof closure has not yet been clearly described in literature thus far. Even though a temporary connection between the pinching off hypophysis and the pharyngeal roof has been reported, clear evidence of development-related cell processes during pharyngeal roof fusion is lacking.

The closure of the Rathke pouch mentioned by Hinrichsen (1993) [9] can thus be described in further detail. Epithelial closure appears to be initiated by induction of apoptotic counts in cells located at the pharyngeal (basal) side of the adenohypophysis. Apoptotic cell death is clearly noticed both histomorphologically and immunohistochemically by evidence of markedly increased amounts of active caspase-3. In contrast to observations of Han et al. (1998) [10] we failed to find apoptotic activities in all adenohypophyseal areas. The epithelial basal closure, therefore, compares to processes occurring during nasolacrimal duct development. The latter closes at the ectodermal surface involving apoptotic processes, while the lumen-directed double-walled epithelial sheet may persist for some time [11].

Regular apoptotic events in the adenohypophysis, e.g. depending upon hormonal influences, were described by several authors. However, all of these studies were confined to adult subjects whose adenohypophysis features fully differentiated cells [12-15]. The embryonic test individuals studied here have not yet arrived at such a stage of maturity.

Impressingly, the apoptotic processes begin long before cell fusion in the pharyngeal roof at a time when the opposite cells to be fused are not yet in contact, with a distance of about 100 μm.

The diencephalon overall features a narrow band of neuroectodermal cells. Distinct proliferation and reinforcement of the neuroectoderm is absent but opposite to the Rathke pouch. This site represents the early neurohypophyseal primordium whose proliferating cells, in contrast to the marginal cells of the Rathke pouch, fail to display a high-prismatic shape (Fig. 2, 5). A direct connection between the neurohypophyseal and adenohypophyseal primordia without interposed mesenchymal cells after Starck (1975) [1] was not confirmed.

## Conclusion

Programmed cell death (apoptosis) plays an important role in embryonic development and tissue homeostasis. In agreement with others our data suggest that a temporally and spatially regulated pattern of apoptosis is also essential for the development of the basis of adenohypophyseal structures. We can clearly show an increasing number of apoptotic events only at the basic fusion sides of the adenohypophysis as well as in the opening region of this organ. Apoptotic destruction of epithelial cells at the basal cones of the adenohypophysis begins even before differentiation of the adenohypophyseal cells and their contact with the neurohypophysis. In early stages of development, thus, apoptotic activity of the adenohypophysis is restricted to the basal areas mentioned. In our test animals, the adenohypophysis develops after closure of the anterior neuroporus. It should be stated that in the top of the developing adenohypophysis no apoptotic cells were detectible. And it is notable that this is a temporary result of the turning of the adenohypophysis out of the pharyngeal roof.

## Competing interests

The authors declare that they have no competing interests.

## Authors' contributions

JW drafted the manuscript, performed the histological investigations. KL helped with the animal trial. AF: helped to the critical review of the manuscript. OD: helped to the critical review of the manuscript, helped to draft the manuscript. JK: helped to the critical review of the manuscript. TG: helped with the histological investigation. PP: performed the surgical procedure.
